# Experiences of Racism in School and Associations with Mental Health, Suicide Risk, and Substance Use Among High School Students — Youth Risk Behavior Survey, United States, 2023

**DOI:** 10.15585/mmwr.su7304a4

**Published:** 2024-10-10

**Authors:** Izraelle I. McKinnon, Kathleen H. Krause, Nicolas A. Suarez, Tiffany M. Jones, Jorge V. Verlenden, Yolanda Cavalier, Alison L. Cammack, Christine L. Mattson, Rashid Njai, Jennifer Smith-Grant, Cecily Mbaka, Jonetta J. Mpofu

**Affiliations:** ^1^Division of Adolescent and School Health, National Center for Chronic Disease Prevention and Health Promotion, CDC, Atlanta, Georgia; ^2^Epidemic Intelligence Service, CDC, Atlanta, Georgia; ^3^Division of Injury Prevention, National Center for Injury Prevention and Control, CDC, Atlanta, Georgia; ^4^Division of Overdose Prevention, National Center for Injury Prevention and Control, CDC, Atlanta, Georgia; ^5^U.S. Public Health Service Commissioned Corps, Rockville, Maryland; ^6^Office of Health Equity, Office of the Director, CDC, Atlanta, Georgia

## Abstract

Racism is a fundamental determinant of health inequities among racial and ethnic groups and is understudied among adolescents. In 2023, the national Youth Risk Behavior Survey questionnaire included an item assessing experiences of racism in the school setting among students in grades 9–12 in the United States. This report estimates the prevalence of students who reported ever having experienced racism in school and compares prevalence by racial and ethnic groups. For each racial and ethnic group, prevalence differences and prevalence ratios were estimated comparing the prevalence of indicators of poor mental health, suicide risk, and substance use among students who reported that they have ever versus never experienced racism in school. In 2023, approximately one in three high school students (31.5%) said that they had ever experienced racism in school. Reported experiences of racism were most prevalent among Asian (56.9%), multiracial (48.8%), and Black or African American (Black) (45.9%) students and least prevalent among White students (17.3%). Black and Hispanic or Latino (Hispanic) students who reported experiencing racism had a higher prevalence of all health risk behaviors and experiences investigated, including indicators of poor mental health, suicide risk, and substance use compared with students of their racial and ethnic group who reported never experiencing racism. Many of these associations were also found among multiracial and White students. Student reports of racism were associated with indicators of mental health and suicide risk among American Indian or Alaska Native (AI/AN) and Asian students. Among students of color, including AI/AN, Asian, Black, Hispanic, and multiracial students, the prevalence of seriously considering and attempting suicide was more than two times higher among students who ever compared with never experienced racism. These findings demonstrate that racism in the school setting is experienced by high school students attending public and private schools and continues to disproportionately affect students of color. Students who reported experiencing racism had a higher prevalence of indicators of poor mental health, suicide risk, and substance use. Schools can incorporate policies and practices to prevent unfair treatment on the basis of race and ethnicity and offer resources to help students cope with these experiences.

## Introduction

Racism, defined as “a system consisting of structures, policies, practices, and norms that assigns value and determines opportunity based on the way people look or the color of their skin,” persists within U.S. society and “is the root cause of many health disparities” (*1*) (https://www.cdc.gov/minorityhealth/racism-disparities/index.html). Associations between self-reported experiences of racism and the social, emotional, mental, and physical health of racial and ethnic communities that have been marginalized are well-documented (*2*). Because experiences of racism can have cumulative effects on health throughout the life course, experiences of racism early in childhood and adolescence can be particularly detrimental (*3*). In 2021, the Adolescent Behaviors and Experiences Survey (ABES) demonstrated that up to one third of all high school students nationwide and more than 50% of Asian, Black or African American (Black), and multiracial high school students in the United States reported that they had ever experienced racism in school (*4*). Typically, high school students spend up to 6 hours per day and 180 days per year in school (*5*), making the school environment an important social structure for adolescents (*6*) where positive or negative experiences with administrators, teachers, and other students can have an impact on their health and well-being. Therefore, continuing to monitor, understand, and address experiences of racism among adolescents in schools and intervening to prevent these experiences and their acute and lasting effects on health is important.

In 2023, for the first time, the national Youth Risk Behavior Survey (YRBS) included a question assessing experiences of racism in school. This is the first report using YRBS data to examine experiences of racism in school and its association with health. The objectives of this report were to describe the prevalence of experiencing racism in school, overall and stratified by race and ethnicity, and to describe associations between experiencing racism in school and mental health, suicide risk, and substance use by racial and ethnic group. Findings from this report can assist public health practitioners, school leaders, teachers, parents, students, and policymakers in understanding the prevalence of racism experienced by students in schools and associations with health risk behaviors. Increased understanding of students’ experience of racism in school and associated health outcomes can provide evidence and guidance for strategies that promote health and well-being for all students.

## Methods

### Data Source

This report includes data from the 2023 YRBS (N = 20,103), a cross-sectional, school-based survey conducted biennially since 1991. Each survey year, CDC collects data from a nationally representative sample of public and private school students in grades 9–12 in the 50 U.S. states and the District of Columbia. Additional information about YRBS sampling, data collection, response rates, and processing is available in the overview report of this supplement (*7*). The prevalence estimates for experiences of racism in school for the study population overall and stratified by sex, race and ethnicity, grade, and sexual identity are available at https://nccd.cdc.gov/youthonline/App/Default.aspx. The full YRBS questionnaire, data sets, and documentation are available at https://www.cdc.gov/yrbs/index.html. Institutional reviews boards at CDC and ICF, the survey contractor, approved the protocol for YRBS. Data collection was conducted consistent with applicable Federal law and CDC policy.*

### Measures

Experience of racism in school was measured by the question, “During your life, how often have you felt that you were treated badly or unfairly in school because of your race or ethnicity?” (never, rarely, sometimes, most of the time, or always). This measure, adapted from the Perceptions of Racism in Children and Youth (PRaCY) scale, has demonstrated validity and reliability among children and youths aged 8–18 years from diverse racial and ethnic backgrounds (*8*). This measure was dichotomized to ever (rarely, sometimes, most of the time, or always) versus never (never) in all analyses. A definition of racism was not given to students when responding to this question.

Health risk behaviors and experiences were investigated in association with experiences of racism (Table 1). Demographic measures included sex, race and ethnicity (American Indian or Alaska Native [AI/AN], Asian, Black or African American [Black], Native Hawaiian or other Pacific Islander [NH/OPI], White, Hispanic or Latino [Hispanic], or multiracial [selected >1 racial category]) (persons of Hispanic or Latino origin might be of any race but are categorized as Hispanic; all racial groups are non-Hispanic), grade (9, 10, 11, and 12), and sexual identity (heterosexual [straight]; lesbian, gay, bisexual, or questioning [I am not sure about my sexual identity/questioning]; or describe identity in some other way [I describe my identity some other way] [LGBQ+]).

**TABLE 1:** Questions, response options, and analytic coding for experiences of racism in school, mental health, suicide risk, and substance use among high school students — Youth Risk Behavior Survey, United States, 2023

Variable	Question	Response option	Analytic coding
**Experiences of racism in school**			
Experienced racism in school	During your life, how often have you felt that you were treated badly or unfairly in school because of your race or ethnicity?	Never, rarely, sometimes, most of the time, or always	Ever: Rarely, sometimes, most of the time, or always Never: Never
**Mental health**			
Current poor mental health	During the past 30 days, how often was your mental health not good? (Poor mental health includes stress, anxiety, and depression.)	Never, rarely, sometimes, most of the time, or always	Yes: Most of the time or always No: Never, rarely, or sometimes
Persistent feelings of sadness or hopelessness	During the past 12 months, did you ever feel so sad or hopeless almost every day for two weeks or more in a row that you stopped doing some usual activities?	Yes or no	Yes: Yes No: No
**Suicide risk**			
Seriously considered attempting suicide	During the past 12 months, did you ever seriously consider attempting suicide?	Yes or no	Yes: Yes No: No
Attempted suicide	During the past 12 months, how many times did you actually attempt suicide?	0 times, 1 time, 2 or 3 times, 4 or 5 times, or ≥6 times	Yes: 1 time, 2 or 3 times, 4 or 5 times, or ≥6 times No: 0 times
**Substance use**			
Current use of any tobacco product	During the past 30 days, on how many days did you A) smoke cigarettes? B) smoke cigars, cigarillos, or little cigars, such as Swisher Sweets, Middleton’s (including Black and Mild), or Backwoods? C) use chewing tobacco, snuff, dip, snus, or dissolvable tobacco products, such as Copenhagen, Grizzly, Skoal, Camel Snus, or Velo Nicotine Lozenges? D) use an electronic vapor product	0 days, 1 or 2 days, 3–5 days, 6–9 days, 10–19 days, 20–29 days, or all 30 days	Yes: 1 or 2 days,3–5 days, 6–9 days, 10–19 days, 20–29 days, or all 30 days for any included substances No: 0 days for all included substances
Current alcohol use	During the past 30 days, on how many days did you have at least one drink of alcohol?	0 days, 1 or 2 days, 3–5 days, 6–9 days, 10–19 days, 20–29 days, or all 30 days	Yes: 1 or 2 days, 3–5 days, 6–9 days, 10–19 days, 20–29 days, or all 30 days No: 0 days
Current marijuana use	During the past 30 days, how many times did you use marijuana?	0 times, 1 or 2 times, 3–9 times, 10–19 times, 20–39 times, or ≥40 times	Yes: 1 or 2 times, 3–9 times, 10–19 times, 20–39 times, or ≥40 times No: 0 times
Current prescription opioid misuse	During the past 30 days, how many times did you take prescription pain medicine without a doctor’s prescription or differently than how a doctor told you to use it?	0 times, 1 or 2 times, 3–9 times, 10–19 times, 20–39 times, or ≥40 times	Yes: 1 or 2 times, 3–9 times, 10–19 times, 20–39 times, or ≥40 times No: 0 times

### Analysis

Prevalence of ever experiencing racism in school was estimated for all students and stratified by race and ethnicity. Within racial and ethnic groups, prevalence was further stratified by sex, grade, and sexual identity. Prevalence ratios (PRs) were estimated comparing the prevalence of experiencing racism by racial and ethnic groups and within them (by sex, grade, and sexual identity), with White students, male students, heterosexual students, and students in grade 9 as the referent group when applicable. Referent groups were those with the lowest prevalence of experiences of racism in school based on 2021 ABES data (White students and male students) (*4*) or referent groups based on common practice with survey data (heterosexual students and students in grade 9). The prevalence of health risk behaviors and experiences for students who reported having ever and never experienced racism in school was calculated for the overall sample and stratified by race and ethnicity. For the overall sample, *t*-tests compared the prevalence of health risk behaviors and experiences among students who ever versus never experienced racism in school. However, within each racial and ethnic group, prevalence differences (PDs) and PRs were estimated comparing the prevalence of health risk behaviors and experiences among students who ever versus never experienced racism in school. All prevalence estimates and measures of association used Taylor series linearization. PDs and PRs were calculated using logistic regression with predicted marginals. Differences detected by *t*-test analyses were considered statistically significant at the p<0.05 level. PRs were considered statistically significant if the 95% CIs did not include 1.0, and p values for PDs were considered statistically significant at the p<0.05 level. Prevalence estimates with denominators <30 were considered statistically unreliable and therefore were suppressed (*7*); thus, associations between racism and health risk behaviors and experiences could not be assessed among NH/OPI students. All analyses were conducted using SAS-callable SUDAAN (version 11.0.4; RTI International) to account for the complex sampling design and weighting.

## Results

Overall, 31.5% of high school students reported having ever experienced racism in school (Table 2). The prevalence of experiencing racism was 56.9% among Asian students, 48.8% among multiracial students, 45.9% among Black students, 39.4% among Hispanic students, 38.0% among AI/AN students, 37.6% among NH/OPI students, and 17.3% among White students. Reports of ever experiencing racism in school were two to three times higher among non-White compared with White students. Among Asian, Black, Hispanic, and multiracial students, the prevalence of ever experiencing racism in school was approximately 1.2 times higher among female students compared with male students (Supplementary Table, https://stacks.cdc.gov/view/cdc/160512). Among Black, Hispanic, and multiracial students, the prevalence of ever experiencing racism in school was approximately 1.2–1.5 times higher among LGBQ+ students compared with heterosexual students.

**TABLE 2:** Prevalence of ever experiencing racism in school among high school students, overall and by race and ethnicity — Youth Risk Behavior Survey, United States, 2023*

Characteristic	Ever experienced racism^†^ %^§^ (95% CI)	PR^¶^ (95% CI)
Race and ethnicity**		
American Indian or Alaska Native	38.0 (27.2–50.1)	2.20^††^ (1.58–3.07)
Asian	56.9 (45.0–68.0)	3.30^††^ (2.57–4.22)
Black or African American	45.9 (37.6–54.4)	2.66^††^ (2.13–3.31)
Native Hawaiian or other Pacific Islander	37.6 (19.6–59.8)	2.18^††^ (1.21–3.94)
White	17.3 (15.1–19.7)	Ref
Hispanic or Latino	39.4 (35.8–43.1)	2.28^††^ (1.95–2.68)
Multiracial	48.8 (42.4–55.3)	2.83^††^ (2.34–3.42)
**Total**	**31.5 (29.0–34.2)**	**—**

**Abbreviations:** PR =prevalence ratio; Ref = referent group.

* N = 20,103 respondents. The total number of students answering each question varied. Data might be missing because 1) the question did not appear in that student’s questionnaire, 2) the student did not answer the question, or 3) the response was set to missing because of an out-of-range response or logical inconsistency. Percentages in each category are calculated on the known data.

^†^ Experiencing racism was categorized as ever for those who responded “rarely,” “sometimes,” “most of the time,” or “always,” and never for those who responded “never.”

^§^ Weighted prevalence estimate.

^¶^ PR comparing the prevalence of ever experienced racism by racial and ethnic group.

** Persons of Hispanic or Latino origin might be of any race but are categorized as Hispanic; all racial groups are non-Hispanic.

^††^ Statistically significant; 95% CIs did not include 1.0.

Among students overall, poor mental health, suicide risk, and substance use were consistently higher among students who reported having ever experienced racism in school compared with students who reported having never experienced racism (Table 3). In analyses stratified by racial and ethnic groups, experiencing racism was associated with health risk behaviors and experiences on both the absolute and relative scale, although patterns and strength of associations varied (Tables 4 and 5).

**TABLE 3:** Prevalence of selected health risk behaviors, by experiences of racism in school among high school students — Youth Risk Behavior Survey, United States, 2023*

Behavior	Ever experienced racism^†^	Never experienced racism^†^
	Prevalence %^§^ (95% CI)	Prevalence %^§^ (95% CI)
**Mental health**		
Current poor mental health	34.8 (31.5–38.2)^¶^	25.9 (24.1–27.8)
Persistent feelings of sadness or hopelessness	54.3 (51.5–57.1)^¶^	33.8 (31.8–35.7)
**Suicide risk**		
Seriously considered attempting suicide	27.8 (25.2–30.6)^¶^	16.8 (15.3–18.4)
Attempted suicide	15.0 (12.9–17.3)^¶^	6.7 (5.8–7.8)
**Substance use**		
Current use of any tobacco product	22.2 (19.9–24.7)^¶^	15.9 (14.2–17.8)
Current alcohol use	26.4 (24.2–28.6)^¶^	20.0 (18.1–22.1)
Current marijuana use	21.8 (19.0–24.8)^¶^	14.5 (13.2–16.0)
Current prescription opioid misuse	8.5 (7.2–10.1)^¶^	2.6 (2.1–3.2)

* N = 20,103 respondents. The total number of students answering each question varied. Data might be missing because 1) the question did not appear in that student’s questionnaire, 2) the student did not answer the question, or 3) the response was set to missing because of an out-of-range response or logical inconsistency. Percentages in each category are calculated on the known data.

^†^ Experiencing racism was categorized as ever for those who responded “rarely,” “sometimes,” “most of the time,” or “always,” and never for those who responded “never.”

^§^ Weighted prevalence estimate.

^¶^ Significantly different from students who report never experiencing racism based on *t*-test analysis with Taylor series linearization (p<0.05).

**TABLE 4:** Prevalence of selected health risk behaviors and experiences of racism in school among high school students, among American Indian or Alaska Native,* Asian,* and Black or African American* high school students — Youth Risk Behavior Survey, United States, 2023^†^

Behavior	American Indian or Alaska Native*				Asian*				Black or African American *			
	Ever experienced racism^§^	Never experienced racism^§^	PR** (95% CI)	PD** (%)	Ever experienced racism^§^	Never experienced racism^§^	PR** (95% CI)	PD** (%)	Ever experienced racism^§^	Never experienced racism^§^	PR** (95% CI)	PD** (%)
	Prevalence %^¶^ (95% CI)	Prevalence %^¶^ (95% CI)			Prevalence %^¶^ (95% CI)	Prevalence %^¶^ (95% CI)			Prevalence %^¶^ (95% CI)	Prevalence %^¶^ (95% CI)		
Current poor mental health	53.3 (33.0–72.5)	25.2 (15.0–39.3)	2.11^††^ (1.14–3.92)	28.0^§§^	27.0 (20.5–34.6)	17.7 (13.0–23.7)	1.52^††^ (1.11–2.09)	9.3^§§^	33.2 (29.4–37.3)	22.3 (17.0–28.6)	1.49^††^ (1.10–2.03)	10.9^§§^
Persistent feelings of sadness or hopelessness	74.7 (60.7–85.0)	44.5 (29.7–60.3)	1.68^††^ (1.14–2.48)	30.2^§§^	43.1 (38.5–47.9)	18.8 (13.3–25.8)	2.30^††^ (1.62–3.26)	24.3^§§^	51.1 (47.0–55.2)	29.9 (26.8–33.2)	1.71^††^ (1.48–1.97)	21.2^§§^
Seriously considered attempting suicide	42.2 (23.3–63.7)	18.6 (10.1–31.8)	2.27^††^ (1.08–4.78)	23.6^§§^	19.7 (16.4–23.5)	6.4 (3.5–11.3)	3.09^††^ (1.69–5.65)	13.3^§§^	27.0 (23.0–31.5)	13.4 (11.6–15.4)	2.02^††^ (1.66–2.44)	13.6^§§^
Attempted suicide	20.4 (11.1–34.5)	7.5 (3.6–14.7)	2.74^††^ (1.23–6.10)	13.0^§§^	11.0 (7.0–17.1)	3.9 (2.1–7.2)	2.81^††^ (1.48–5.35)	7.1^§§^	15.2 (12.5–18.5)	4.7 (3.3–6.6)	3.25^††^ (2.24–4.71)	10.5^§§^
Current use of any tobacco product	45.5 (25.1–67.6)	25.5 (15.1–39.7)	1.79 (0.91–3.49)	20.0	7.3 (4.6–11.5)	7.1 (2.7–17.6)	1.03 (0.41–2.59)	0.2	21.2 (16.2–27.3)	13.1 (11.0–15.5)	1.62^††^ (1.27–2.06)	8.1^§§^
Current alcohol use	31.9 (20.9–45.3)	22.3 (11.7–38.4)	1.43 (0.77–2.64)	9.6	14.1 (10.0–19.6)	10.7 (8.0–14.4)	1.31 (0.86–2.01)	3.4	22.7 (18.2–28.0)	12.3 (10.0–14.9)	1.85^††^ (1.38–2.49)	10.4^§§^
Current marijuana use	32.0 (18.7–49.0)	21.3 (12.0–34.7)	1.50 (0.72–3.15)	10.7	7.5 (4.5–12.4)	4.9 (1.8–12.6)	1.55 (0.54–4.42)	2.7	24.1 (18.9–30.1)	12.8 (10.2–15.9)	1.88^††^ (1.43–2.48)	11.3^§§^
Current prescription opioid misuse	1.4 (0.6–3.4)	1.3 (0.6–2.8)	1.02 (0.37–2.83)	0.0	6.0 (2.8–12.6	2.9 (1.0–8.3)	2.05 (0.75–5.61)	3.1	6.8 (4.8–9.6)	3.6 (2.5–5.1)	1.92^††^ (1.20–3.07)	3.3^§§^

**Abbreviations:** PD = prevalence difference; PR = prevalence ratio.

* Persons of Hispanic or Latino origin might be of any race but are categorized as Hispanic; all racial groups are non-Hispanic.

^†^ N = 20,103 respondents. The total number of students answering each question varied. Data might be missing because 1) the question did not appear in that student’s questionnaire, 2) the student did not answer the question, or 3) the response was set to missing because of an out-of-range response or logical inconsistency. Percentages in each category are calculated on the known data.

^§^ Experiencing racism was categorized as ever for those who responded “rarely,” “sometimes,” “most of the time,” or “always,” and never for those who responded “never.”

^¶^ Weighted prevalence estimate.

** PR and PD comparing the prevalence of health risk behaviors and experiences, by ever versus never experiencing racism.

^††^ Statistically significant; 95% CIs did not include 1.0.

^§§^ p value for prevalence difference based on *t*-test analysis with Taylor series linearization (<0.05).

**TABLE 5:** Prevalence of selected health risk behaviors and experiences of racism in school among high school students, among Hispanic or Latino, multiracial,* and White* high school students — Youth Risk Behavior Survey, United States, 2023^†^

Behavior	Hispanic or Latino*				Multiracial*				White*			
	Ever experienced racism^§^	Never experienced racism^§^	PR** (95% CI)	PD** (%)	Ever experienced racism^§^	Never experienced racism^§^	PR** (95% CI)	PD** (%)	Ever experienced racism^§^	Never experienced racism^§^	PR** (95% CI)	PD** (%)
	Prevalence %^¶^ (95% CI)	Prevalence %^¶^ (95% CI)			Prevalence %^¶^ (95% CI)	Prevalence %^¶^ (95% CI)			Prevalence %^¶^ (95% CI)	Prevalence %^¶^ (95% CI)		
Current poor mental health	36.1 (31.3–41.2)	19.8 (17.3–22.5)	1.83^††^ (1.59–2.10)	16.3^§§^	39.0 (32.8–45.5)	20.6 (16.1–26.0)	1.89^††^ (1.48–2.41)	18.4^§§^	36.1 (29.9–42.8)	30.5 (27.8–33.2)	1.18 (0.97–1.44)	5.6
Persistent feelings of sadness or hopelessness	58.5 (54.7–62.2)	32.5 (29.6–35.5)	1.80^††^ (1.64–1.99)	26.0^§§^	53.3 (48.2–58.3)	30.7 (23.4–39.1)	1.73^††^ (1.38–2.18)	22.6^§§^	55.2 (50.7–59.6)	36.2 (33.7–38.9)	1.52^††^ (1.39–1.66)	18.9^§§^
Seriously considered attempting suicide	26.3 (23.7–29.1)	12.9 (10.8–15.3)	2.04^††^ (1.70–2.45)	13.5^§§^	30.4 (24.4–37.1)	14.0 (10.1–19.0)	2.17^††^ (1.56–3.02)	16.4^§§^	32.3 (26.2–39.0)	19.9 (18.0–21.9)	1.62^††^ (1.36–1.94)	12.4^§§^
Attempted suicide	16.7 (13.9–19.9)	7.0 (5.5–9.0)	2.37^††^ (1.81–3.09)	9.6^§§^	15.3 (11.1–20.8)	7.6 (4.5–12.6)	2.02^††^ (1.13–3.63)	7.8^§§^	12.9 (9.5–17.3)	7.0 (5.8–8.5)	1.84^††^ (1.35–2.51)	5.9^§§^
Current use of any tobacco product	22.8 (19.2–26.9)	13.0 (10.9–15.6)	1.75^††^ (1.44–2.13)	9.8^§§^	24.3 (18.7–30.9)	14.9 (10.4–20.9)	1.62^††^ (1.12–2.35)	9.3^§§^	26.5 (21.9–31.7)	18.2 (15.6–21.2)	1.45^††^ (1.23–1.72)	8.2^§§^
Current alcohol use	27.8 (24.1–31.9)	15.4 (13.0–18.2)	1.80^††^ (1.45–2.24)	12.4^§§^	24.5 (20.7–28.7)	20.8 (16.0–26.5)	1.18 (0.88–1.57)	3.7	32.0 (27.6–36.7)	24.2 (21.1–27.5)	1.32^††^ (1.14–1.53)	7.8^§§^
Current marijuana use	22.4 (18.6–26.7)	13.3 (11.0–16.0)	1.68^††^ (1.37–2.06)	9.1^§§^	25.9 (20.8–31.7)	15.6 (11.8–20.3)	1.66^††^ (1.27–2.17)	10.3^§§^	21.9 (16.9–27.8)	15.9 (13.8–18.4)	1.37^††^ (1.12–1.68)	5.9^§§^
Current prescription opioid misuse	9.8 (7.2–13.1)	3.0 (2.1–4.4)	3.23^††^ (1.90–5.47)	6.7^§§^	8.8 (5.6–13.5)	3.1 (1.7–5.6)	2.79^††^ (1.47–5.29)	5.6^§§^	8.0 (5.6–11.4)	2.1 (1.6–2.9)	3.76^††^ (2.44–5.79)	5.9^§§^

**Abbreviation:** PD = prevalence difference; PR = prevalence ratio.

* Persons of Hispanic or Latino origin might be of any race but are categorized as Hispanic; all racial groups are non-Hispanic.

^†^ N = 20,103 respondents. The total number of students answering each question varied. Data might be missing because 1) the question did not appear in that student’s questionnaire, 2) the student did not answer the question, or 3) the response was set to missing because of an out-of-range response or logical inconsistency. Percentages in each category are calculated on the known data.

^§^ Experiencing racism was categorized as ever for those who responded “rarely,” “sometimes,” “most of the time,” or “always,” and never for those who responded “never.”

^¶^ Weighted prevalence estimate.

** PR and PD comparing the prevalence of health risk behaviors and experiences, by ever versus never experiencing racism.

^††^ Statistically significant; 95% CIs did not include 1.0.

^§§^ p value for prevalence difference based on *t*-test analysis with Taylor series linearization (<0.05).

AI/AN, Asian, Black, Hispanic, and multiracial students who reported having ever (versus never) experienced racism in school had a higher prevalence of current poor mental health and persistent feelings of sadness or hopelessness. Among White students, persistent feelings of sadness or hopelessness, but not poor mental health, was significantly higher among those who reported having ever (versus never) experienced racism. Across all racial and ethnic minority groups, the prevalence of seriously considering attempting suicide and suicide attempts during the past year was higher among those who reported having ever (versus never) experienced racism. Among Black, Hispanic, and White students, the prevalence of current use of any tobacco product, alcohol, marijuana, and prescription opioid misuse was higher among students who reported having ever (versus never) experienced racism in school. Multiracial students who reported having experienced racism in school also had a higher prevalence of current use of any tobacco product, marijuana, and prescription opioid misuse.

## Discussion

In 2023, approximately one in three high school students across the United States reported that they had ever experienced racism in school, described as unfair treatment in school because of their race or ethnicity. Experiences of racism were two to three times more prevalent among students of color (i.e., AI/AN, Asian, Black, Hispanic, multiracial, and NH/OPI students) compared with White students, with Asian students reporting the highest prevalence of having ever experienced racism. The high prevalence of experiences of racism and associations between racism and health risk behaviors among Asian students in this report align with findings from the 2021 ABES, which used the same question to assess exposure to racism that was used in the 2023 YRBS to provide nationally representative estimates of experiences of racism among high school students during the COVID-19 pandemic (*4*). In 2023, the U.S. Commission on Civil Rights provided a report on the Federal response to anti-Asian racism in the United States, documenting the increasing prevalence of anti-Asian discrimination since the COVID-19 pandemic (https://www.usccr.gov/files/2023-10/fy-2023-se-report.pdf). The report also describes Federal initiatives to increase awareness, prevention, and reporting of anti-Asian discrimination. For example, in 2021, the Office of Juvenile Justice and Delinquency Prevention under the U.S. Department of Justice launched a national initiative to prevent youth hate crimes and identity-based bullying among Asian American and Pacific Islander students (https://ojjdp.ojp.gov/programs/preventing-youth-hate-crimes-bullying-initiative#about-the-initiative). In addition, the U.S. Department of Justice Civil Rights Division and the U.S. Department of Education Office for Civil Rights fact sheet on confronting COVID-19–related harassment in schools provides multiple action steps families can take to work with schools to respond to anti-Asian discrimination among students (https://www.justice.gov/crt/page/file/1392041/dl?inline). Although prevalence estimates of experiences of racism in school were lower across racial and ethnic groups (with the exception of AI/AN students) in the 2023 YRBS compared with the 2021 ABES (*4*), direct comparisons between YRBS and ABES results cannot be made because of differences in methodology (e.g., the ABES was self-administered by students online in various settings).

Previous studies also have found associations between experiences of racism among adolescents and mental health, suicide risk, and substance use. For example, a 2018 meta-analytic review of studies on racial and ethnic discrimination during adolescence demonstrated associations with depressive symptoms and substance use, with the majority of studies using a general measure of experiences of racism that did not specify setting or perpetrator (*9*). Most studies examining associations between self-reported experiences of racism and health among adolescents have focused on mental health (*2*). Strong associations between racism and suicide risk found in this study align with findings from a recent study using ABES data. Using data from students of color, in unadjusted models, students who sometimes, most of the time, or always experienced racism in school had 3.38 times higher odds of seriously considering suicide and 3.87 times higher odds of attempting suicide during the past 12 months compared with students who never experienced racism (*10*). Whereas the current study included students rarely experiencing racism in the comparison group against students never experiencing racism, more frequent experiences of racism might have stronger associations with suicide risk among students (*10*).

Students might be experiencing racism in school because of discrimination and bias that are embedded within current school policies and practices (e.g., disciplinary practices) or as a result of interactions with students, teachers, or administrators and other staff members (*11*). Schools can implement and maintain policies and practices to prevent and to address experiences of racism occurring in school. Schools can provide professional development to teachers, administrators, and other school staff members to increase their awareness of racism in schools, including personal implicit and explicit biases that might affect their treatment of students, and build skills to intervene when they witness racism (*12*). For example, the San Diego Unified School District has developed the Equity Collective (https://www.sdusdequity.com/whoweare) to provide professional development opportunities for staff members to increase their understanding of how equity, bias, and oppression affect social, emotional, academic, and behavioral outcomes among students. Such professional development can also equip staff members to provide school and community-based resources to support students who have experienced racism (e.g., referrals for mental health treatment and resources for coping behaviors). Training staff members to adopt discipline and teaching strategies that reflect cultural competency and cultural humility might serve to create healthier socioemotional environments for all students and faculty, regardless of race and ethnicity (*13*).

Schools also can promote policies and practices to prevent systemic inequities in treatment that disproportionately affect the mental health and well-being of students of color. For example, in 2023, the U.S. Department of Justice and the U.S. Department of Education released a resource on confronting racial discrimination in student discipline, which documents multiple investigations of discriminatory practices in student discipline on the basis of race and ethnicity and provides proactive solutions to reduce reliance on discipline systems that might be implicitly or explicitly biased to disproportionately affect students from marginalized groups and their mental health (https://www2.ed.gov/about/offices/list/ocr/docs/tvi-student-discipline-resource-202305.pdf). Schools can implement culturally responsive positive behavior interventions and supports as well as participatory problem-solving approaches that engage families and communities (*14*). Seattle Public Schools created the Office of African American Male Achievement (AAMA) in 2019. AAMA works with students, families, and educators to promote school and community environments that promote success of Black boys and teens by cultivating their strengths instead of approaching student behavior from a deficit model, using a framework of systems change rather than student intervention. In addition, schools across the United States have implemented student-led affinity and intersectional groups for students identifying with racial and ethnic groups who have been marginalized (*15*). Such groups can provide students with an environment to discuss their experiences and develop coping and self-regulation skills, as well as develop a positive social identity and historical and cultural knowledge that affirms and accurately describes their identity. Schools also can ensure access to certified school counselors and social workers as a structural intervention to address harms from racism. CDC provides resources for action to promote mental well-being and prevent mental distress and suicide risk among children and adolescents and strategies that can help mitigate the impact of racism, including Promoting Mental Health and Well-Being in Schools: An Action Guide for School and District Leaders (https://www.cdc.gov/healthyyouth/mental-health-action-guide/index.html), the Suicide Prevention Resource for Action (https://www.cdc.gov/suicide/pdf/preventionresource.pdf), and the Drug-Free Communities Support Program (https://www.cdc.gov/overdose-prevention/php/drug-free-communities/index.html).

## Limitations

General limitations for the YRBS are available in the overview report of this supplement (*7*). The findings in this report are subject to at least three additional limitations. First, the variables included in this study reflect different points in time, including a lifetime experience of racism in school compared with health risk behaviors occurring during the past 30 days (e.g., poor mental health and substance use) or the past 12 months (e.g., feelings of sadness or hopelessness or suicide risk). The temporal ordering of when students experienced racism in relation to the health risk behaviors and experiences included in this report cannot be determined because data were collected through a cross-sectional survey and causality between variables cannot be determined. Second, the experience of racism is measured as a single item and might not capture the complexity of all racial and ethnic populations’ cultural and structural experiences of racism (*2*). Finally, further disaggregation of students by specific racial and ethnic combinations in future studies might provide additional important insights that are diluted when using one category for students selecting more than one racial or ethnic category. For example, students who selected more than one racial category were categorized as multiracial, and students selecting Hispanic ethnicity were categorized as Hispanic whether they selected one or more racial categories.

## Future Directions

The added measure assessing racism in school in the 2023 YRBS questionnaire enables CDC to monitor these experiences among high school students over time. Future studies can examine the relation between experiences of racism in school and other health risk behaviors not included in this report (e.g., poor sleep, physical activity, and dietary behaviors). In-depth studies examining heterogeneity within groups are warranted, as experiences of racism were found to be more prevalent among Asian, female, and LBGQ+ students. For example, although in general, Asian students have a low prevalence of many risk behaviors (https://www.cdc.gov/healthyyouth/data/yrbs/pdf/YRBS_Data-Summary-Trends_Report2023_508.pdf), a majority of Asian students reported experiencing racism at school in this report and others (*4*). In addition, future studies can investigate additional individual, school, and structural-level factors that might contribute to experiences of racism among students, as well as those that might buffer the negative health behaviors and experiences associated with racism.

In future practice, school districts might consider interventions that create safe and supportive environments by promoting school culture, conditions, and competencies that support equity and anti-racism, as well as healing from experiences of racism. Another important consideration is systems-level changes to policies and practices in schools, such as assessing discipline practices that contribute to unfair treatment, supporting positive identity development, and ensuring access to mental health–related resources (e.g., certified school counselors and social workers).

## Conclusion

The findings in this report characterize associations between experiencing racism in school, poor mental health, substance use, and suicide risk for high school students from all racial and ethnic groups. Experiences of racism in school were two to three times more prevalent among AI/AN, Asian, Black, Hispanic, multiracial, and NH/OPI students compared with White students. Such findings highlight the potential benefits of school-based policies and practices that address negative experiences on the basis of race and ethnicity in school. Schools can promote connections to foster positive experiences for all students, including those who have experienced racism. By working to prevent racism in school, schools can serve as a safe and supportive place for all students.

## Footnotes

* 45 C.F.R. part 46.114; 21 C.F.R. part 56.114.
